# The Effect of Adding Biological Factors to the Decision-Making Process for Spinal Metastasis of Non-Small Cell Lung Cancer

**DOI:** 10.3390/jcm10051119

**Published:** 2021-03-08

**Authors:** Hyoungmin Kim, Sam Yeol Chang, Jongyeon Son, Sujung Mok, Sung Cheol Park, Bong-Soon Chang

**Affiliations:** Department of Orthopedic Surgery, Seoul National University Hospital, 101 Daehangno, Jongno-gu, Seoul 03080, Korea; hmkim21@gmail.com (H.K.); 2001jyson@hanmail.net (J.S.); charisma9025@naver.com (S.M.); neoz0708@gmail.com (S.C.P.); bschang@snu.ac.kr (B.-S.C.)

**Keywords:** spinal metastasis, non-small cell lung cancer, decompression, survival, prognosis, epidermal growth factor receptor, Uno’s C-index, New England Spinal Metastasis Score

## Abstract

Molecular target therapies have markedly improved the survival of non-small cell lung cancer (NSCLC) patients, especially those with epidermal growth factor receptor (EGFR) mutations. A positive EGFR mutation is even more critical when the chronicity of spinal metastasis is considered. However, most prognostic models that estimate the life expectancy of spinal metastasis patients do not include these biological factors. We retrospectively reviewed 85 consecutive NSCLC patients who underwent palliative surgical treatment for spinal metastases to evaluate the following: (1) the prognostic value of positive EGFR mutation and the chronicity of spinal metastasis, and (2) the clinical significance of adding these two factors to an existing prognostic model, namely the New England Spinal Metastasis Score (NESMS). Among 85 patients, 38 (44.7%) were EGFR mutation-positive. Spinal metastasis presented as the initial manifestation of malignancy in 58 (68.2%) patients. The multivariate Cox proportional hazard model showed that the chronicity of spinal metastasis (hazard ratio (HR) = 1.88, *p* = 0.015) and EGFR mutation positivity (HR = 2.10, *p* = 0.002) were significantly associated with postoperative survival. The Uno’s C-index and time-dependent AUC 6 months following surgery significantly increased when these factors were added to NESMS (*p* = 0.004 and *p* = 0.022, respectively). In conclusion, biological factors provide an additional prognostic value for NSCLC patients with spinal metastasis.

## 1. Introduction

Lung cancer is the most commonly diagnosed malignancy and accounts for approximately 25% of cancer deaths in men and women [1]. The spinal column is the most frequent site for the extrapulmonary metastasis of non-small cell lung cancer (NSCLC), which accounts for 80–85% of lung cancer cases [2]. The lung is also the most common location for primary cancer when a patient presents with spinal metastasis as an initial manifestation of the disease [3]. The incidence of spinal metastasis associated with NSCLC is increasing because of improved survival in these patients based on recent advancements in systemic treatment for NSCLC, such as tyrosine kinase inhibitors (TKIs) for epidermal growth factor receptor (EGFR) mutations [4,5]. Improved survival and increased incidence of spinal metastasis in NSCLC patients render surgical treatment and related decision-making processes for spinal metastasis more important.

Numerous decision-making systems or prognostic models have been introduced to estimate the remaining life expectancies and to suggest appropriate treatment options for patients with spinal metastasis [6]. Authors have used evolving methodologies, such as machine-learning algorithms, to develop a novel prognostic model for spinal metastasis [7]. These models are based on the prognostic factors significantly associated with patient survival in multivariate logistic or proportional hazards regression analyses [8]. Among these factors, the anatomical site for a primary cancer is the most significant prognostic factor, and is included in all models [9]. However, recent advances in tumor genetics suggest that a simple stratification of primary cancer by the anatomical site is insufficient [10]. Given the extensive evidence in the literature that molecular target therapies significantly improve survival in patients with certain mutations [11], genetic subtype analysis should also be considered when predicting survival in patients with spinal metastasis.

Another biological factor that should be considered in survival prediction is the chronicity of spinal metastasis. Several authors have reported that patients with spinal metastasis at the initial presentation of malignancy (synchronous metastasis) survive longer than those diagnosed with spinal metastasis later during treatment (metachronous metastasis) [3,12]. The development of resistance to previous systemic treatment and the availability of further systemic treatment options have been suggested as potential reasons for the difference in prognosis [13].

The New England Spinal Metastasis Score (NESMS) was recently introduced as a novel prognostic model for patients with spinal metastasis [14]. The NESMS consists of a modified Bauer score component, ambulatory function, and serum albumin (Table 1). The developers of NESMS prospectively validated the system in their following study [15]. However, even the recently developed NESMS system does not consider previously described biological factors when stratifying primary cancer and predicting survival. Therefore, we conducted this study to evaluate the effect of adding biological factors to a validated prognostic model for spinal metastasis—the NESMS. Although multiple prognostic models are available, from conventional scoring systems to novel machine-learning-based models, we chose NESMS because, to the best of our knowledge, it is thus far the only model validated using a well-designed prospective investigation with appropriate power [15].

## 2. Materials and Methods

**Figure 1:**
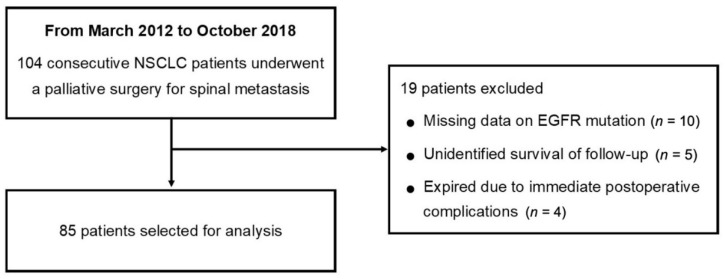
Flowchart for patient selection. (Abbreviations: NSCLC, non-small cell lung cancer; EGFR, epidermal growth factor receptor).

Consecutive patients who underwent palliative surgical treatment for spinal metastasis of lung adenocarcinoma between March 2012 and October 2018 at the authors’ institution were included in the current retrospective study. We included only patients who were biopsy-proven to have adenocarcinoma of the lung and underwent EGFR mutation analysis. Exclusion criteria were as follows: (1) missing data on EGFR mutation analysis results, (2) follow-up period of less than 12 months or unidentified survival period, and (3) patients who died within 2 weeks following surgery due to immediate postoperative complications (Figure 1). The current retrospective study obtained ethical approval and a waiver of informed consent from the institutional review board (IRB No. 2009-060-1155).

Surgeries for NSCLC patients with spinal metastasis were performed based on the decisions made during a weekly multidisciplinary tumor board meeting consisting of medical and radiation oncologists, orthopedic and neuro-surgeons, diagnostic radiologists, and pathologists. In general, surgical treatment was considered for patients who were anticipated to have a postoperative survival period longer than 6 months. Surgical indications included (1) metastatic spinal cord compression and (2) spinal instability causing pain that was uncontrolled by medications or radiotherapy. Three different surgeons from the Department of Orthopedic Surgery operated on these patients. We performed all surgeries for palliation.

Patient information was retrieved from electronic medical records and was retrospectively reviewed. Regarding NSCLC and spinal metastasis; we identified the chronicity of spinal metastasis and the positivity of EGFR mutation as primary dependent variables. Spinal metastasis diagnosed at the initial presentation of NSCLC was referred to as synchronous metastasis, and spinal metastasis diagnosed during the course of NSCLC treatment was referred to as metachronous metastasis. Analysis for EGFR mutation was performed using either direct DNA sequencing analysis or peptide nucleic acid (PNA)-mediated real-time polymerase chain reaction (PCR) clamping analysis [16]. Information on pre- and post-operative systemic treatment regimens, including conventional cytotoxic chemotherapy and target therapies, such as TKIs, were also collected. To evaluate the patients’ preoperative status, we assessed the preoperative ambulatory status and serum albumin, and applied the NESMS using these variables (Table 1). Preoperative serum albumin within 1 week before surgery and preoperative ambulatory status, which was routinely recorded 1 day before surgery, were selected for the preoperative evaluation. Postoperative survival, defined as the time interval between spinal surgery and either death or the last follow-up, was identified as the primary outcome. Patients’ survival beyond 6 months postoperatively was considered the secondary outcome.

Survival probability was estimated using the Kaplan–Meier method (product-limit estimator). The Cox proportional hazard model was applied to develop a prognostic model, and the proportion hazard assumption was checked using log–log plots and the time-by-covariate interaction for each predictor. The Uno’s C-index and time-dependent area under the curve (AUC) 6 months postoperatively were utilized to evaluate the discrimination and prediction ability of the NESMS, and the effect of adding two biological factors (chronicity of spinal metastasis and EGFR mutation positivity) into the NESMS. *p*-values were adjusted using the Bonferroni method. All statistical analyses were performed using SAS system for Windows, version 9.4 (SAS Institute, Cary, NC, USA) and R software version 3.6.1 (R Foundation for Statistical Computing, Vienna, Austria). *p*-values less than 0.05 were considered statistically significant.

## 3. Results

Between March 2012 and October 2018, a total of 104 NSCLC patients received palliative surgery for spinal metastasis at the authors’ institution. Among these patients, 19 were excluded from the analysis for the following reasons: (1) ten due to missing data on EGFR mutation analysis results, (2) five with an unidentified survival period or death, and (3) four who died within two weeks after surgery due to immediate postoperative complications (two pneumonia, one cardiac arrest, and one disseminated intravascular coagulation due to massive bleeding; Figure 1). As a result, 85 patients (58 males and 27 females) with a mean age of 60.9 (range, 32–81) years were analyzed in the current study. The characteristics of the study population are described in Table 2.

**Figure 2:**
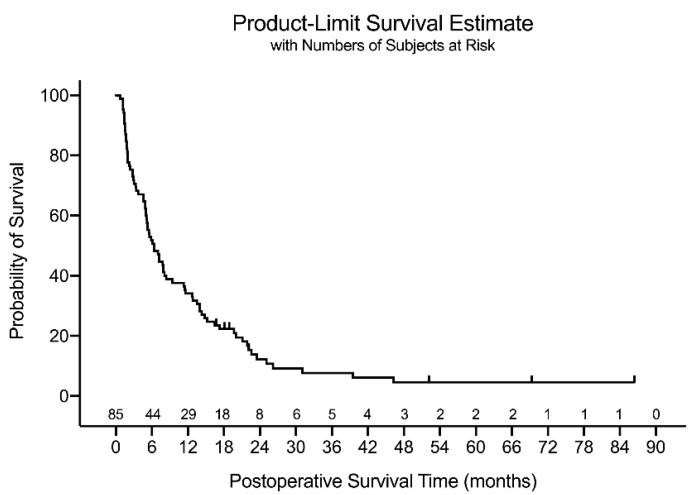
The Kaplan–Meier estimator graph for the total cohort.

**Figure 3:**
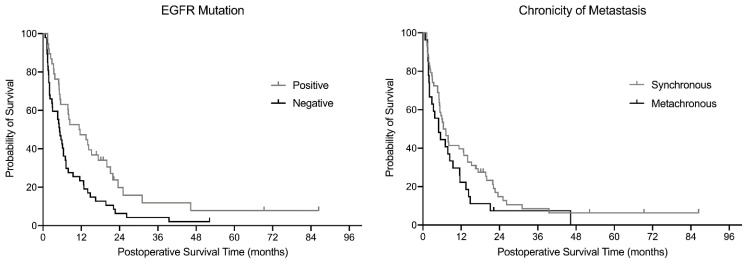
Comparison of the Kaplan–Meier curve stratified by the biological factors.

Seven patients were alive at the last follow-up, with a minimum follow-up period of 12 months, and the remaining 78 died during follow-up. The median postoperative survival period estimated by the Kaplan–Meier estimator was 6.4 months for the entire cohort (*n* = 85; Figure 2). Patients with a positive EGFR mutation had a significantly prolonged survival (*p* = 0.007), and those with synchronous metastasis tended to have longer survival (*p* = 0.101) than their counterparts in the log-rank test (Figure 3). According to the multivariate Cox proportional hazard model, the chronicity of spinal metastasis (hazard ratio (HR) = 1.88 (95% CI: 1.13. 3.12), *p* = 0.015), and EGFR mutation positivity (HR = 2.10 (95% CI: 1.30, 3.38), *p* = 0.002) were significantly associated with postoperative survival (Table 3). All predictors satisfied the proportional hazard assumption.

The Uno’s C-index (discrimination ability) of NESMS was improved from 0.59 (95% CI: 0.54–0.65) to 0.62 (95% CI: 0.56–0.69), 0.64 (95% CI: 0.58–0.71), and 0.67 (95% CI: 0.61–0.74) when the chronicity of spinal metastasis, the EGFR mutation positivity, and both factors were added to the NESMS, respectively (Table 4). The improvement was statistically significant when the EGFR mutation positivity alone (adjusted *p* = 0.019) and both factors (adjusted *p* = 0.004) were added to the NESMS. The time-dependent AUC for predicting survival beyond 6 months postoperatively also increased from 0.63 (95% CI: 0.53–0.74) to 0.73 (95% CI: 0.64–0.82) when the two biological factors were added to the NESMS (adjusted *p* = 0.022; Table 5).

## 4. Discussion

In the late 1990s, gefitinib, an oral EGRF TKI, was introduced as a molecular target therapy for NSCLC patients. A few years later, researchers identified EGFR mutations in NSCLC patients sensitive to gefitinib. Since then, genetic mutation analyses and corresponding molecular target therapies have been game-changers in the management of NSCLC, improving the survival of patients with EGFR mutations [11]. Several previous studies have reported the clinical effects of EGFR mutation positivity and TKIs in NSCLC patients with skeletal [17] and spinal metastasis [18]. In the current study, patients with a positive EGFR mutation showed a significantly prolonged postoperative survival period compared to the EGFR mutation-negative group. The EGFR mutation positivity also significantly improved the discrimination (Uno’s C-index) and prediction ability (time-dependent AUC at 6 months postoperatively) of a novel prognostic model—the NESMS. These results signify the importance of considering biological profiles in the decision-making process for spinal metastasis.

The timing of diagnosis of spinal metastasis, or the chronicity of spinal metastasis, was considered an additional biological factor in this study, which was significantly associated with postoperative survival. In previous studies, not only postoperative survival but also overall survival, was prolonged in patients with spinal metastasis as the initial manifestation of malignancy (synchronous metastasis) [3,12]. From the standpoint of tumor genetics, these findings can be related to the acquired resistance to first-line (first and second generation) TKIs. Common mechanisms for acquired resistance to TKIs, which usually develop within 12 months after TKI usage [13], are mutations in 20 exons (threonine-to-methionine substitution on codon 790, T790M) and MET oncogene amplification [19,20].

**Figure 4:**
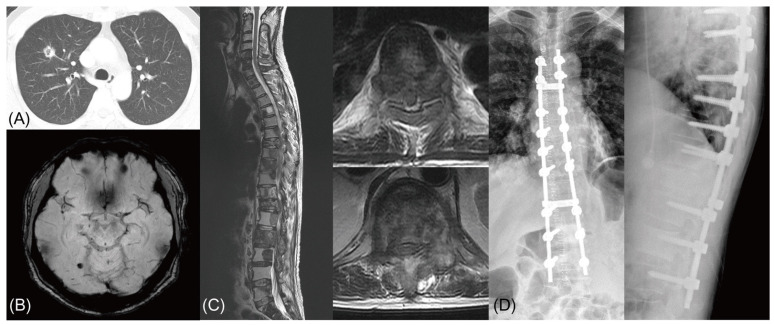
Comparison of Kaplan–Meier curve stratified by the biological factors. An illustrative case of acquired resistance to tyrosine kinase inhibitor (TKI) in an epidermal growth factor receptor (EGFR) mutation-positive non-small cell lung cancer (NSCLC) patient. (**A**,**B**) A 53 years-old male with lung adenocarcinoma in right upper lobe. EGFR mutation analysis from the lung specimen showed a microdeletion mutation in exon 19. (**C**) After 2 years of systemic treatment with multiple regimens including TKI (gefitinib), the patient was diagnosed with multiple spinal metastasis with spinal cord compression at T7 and T12. (**D**) The patient underwent a palliative decompression and stabilization, and EGFR mutation analysis from a spine specimen revealed a missense mutation of EGFR gene exon 20 (T790M). The patient expired 4 months postoperatively due to disease progression.

In our series, 7 (18.4%) of the 38 patients in the EGFR mutation-positive group showed a mutation in exon 20 (T790M) later in their disease course, which was not present in the initial molecular analysis. Five of these seven patients had metachronous spinal metastasis, and their exon 20 mutations were found in specimens obtained from the spine surgery. For these patients, third generation TKI (simertinib) or cytotoxic chemotherapy was considered after spinal surgery, and a shorter life expectancy was anticipated. This effect of acquired resistance to a TKI in metachronous metastasis patients was reflected in our finding that the time-dependent AUC 6 months postoperatively was significantly increased when both factors (EGFR mutation and chronicity) were added to the prognostic model (*p* = 0.022) and not when only EGFR mutation positivity was added (*p* = 0.096). As not all patients in our series underwent additional biopsies and molecular analyses during their disease course, the exact number of patients with acquired resistance to TKI in the metachronous metastasis group cannot be derived. Nevertheless, acquired resistance to TKIs can be associated with shortened survival in metachronous metastasis patients, and therefore, the chronicity of spinal metastasis should be considered as a significant biological factor (Figure 4).

We examined the discrimination and prediction ability of the NESMS, a novel and prospectively validated prognostic model, in this study (Table 1). In this system, the primary tumor is stratified according to the modified Bauer score. As all patients in our series had lung adenocarcinoma, the modified Bauer score was 0 for all patients. Therefore, after eliminating the most significant factor from the NESMS, the remaining factors for the decision-making process are ambulatory function and serum albumin. In this setting, if there are two different NSCLC patients with ambulatory status and serum albumin falling into the same category, the decisions for two patients would be the same according to the NESMS, even if the two have significantly different biological profiles (e.g., synchronous metastasis with a positive EGFR mutation versus metachronous metastasis without EGFR mutation). This novel “classification-based” decision-making system, the NESMS, may be useful and straightforward when all spinal metastasis patients with diverse primary cancers are combined; however, its discrimination ability seems to be significantly limited for individual cancers.

We examined the discrimination and prediction ability of the NESMS, a novel and prospectively validated prognostic model, in this study (Table 1). In this system, the primary tumor is stratified according to the modified Bauer score. As all patients in our series had lung adenocarcinoma, the modified Bauer score was 0 for all patients. Therefore, after eliminating the most significant factor from the NESMS, the remaining factors for the decision-making process are ambulatory function and serum albumin. In this setting, if there are two different NSCLC patients with ambulatory status and serum albumin falling into the same category, decisions for two patients would be the same according to the NESMS, even if the two have significantly different biological profiles (e.g., synchronous metastasis with a positive EGFR mutation versus metachronous metastasis without EGFR mutation). This novel “classification-based” decision-making system, the NESMS, may be useful and straightforward when all spinal metastasis patients with diverse primary cancers are combined; however, its discrimination ability seems to be significantly limited for individual cancers.

It is obvious that a prognostic model’s performance will improve if more prognostic factors are added to it. However, adding too many factors can make a prognostic model complicated and difficult to use in the clinical setting. Therefore, it is essential to prioritize prognostic factors according to their weights in multivariate logistic or proportional hazard regression analyses. Factors with higher odds or hazard ratios should be incorporated into the system. In our study, a multivariate Cox proportional hazard model (backward stepwise with likelihood ratio test) yielded a higher hazard ratio for EGFR mutation positivity (HR = 2.27 (95% CI: 1.41, 3.66), *p* = 0.001) than ambulatory status (HR = 2.26 (95% CI: 1.29, 3.95), *p* = 0.004) and serum albumin (HR = 1.71 (95% CI: 0.96, 3.02), *p* = 0.068), which are the main components of the NESMS. These results also emphasize the importance and necessity of adding biological factors as modifiers in the decision-making systems for spinal metastasis.

Among the various decision-making systems reported in the literature, there have been efforts to incorporate biological factors into these systems. In 2014, Katagiri et al. introduced a revised version of their prognostic system for spinal metastasis, in which the application of molecular target therapy was considered when stratifying the patient’s primary tumor [10]. In their system, lung cancer treated with molecular target therapy was classified as a moderate-growth tumor, while lung cancer without available molecular target therapy was classified as a rapid-growth tumor. Efforts to incorporate biological factors into decision-making systems, as shown in the revised Katagiri system, are anticipated to be the future trends in the management of spinal metastases.

In this study, we stratified patients by EGFR mutation positivity rather than by the treatment they received (e.g., TKI versus platinum-based chemotherapy), as in a previous study [18]. The most important reason for choosing this categorization is that the EGFR mutation profile, rather than the type of postoperative systemic treatment the patient will receive after surgery, is more available at the time of decision-making for spinal metastasis surgeries. As the purpose of this study was to verify the prognostic value of biological factors and not to compare the treatment outcomes, our categorization seems to be more appropriate. Another reason is the diversity of systemic treatment that a patient with NSCLC receives after surgery, as well as the start point and duration of these treatments. In our series, 41 (48.2%) patients received a combination of molecular target therapy and cytotoxic chemotherapy, whereas only 14 (16.5%) received molecular target therapy alone postoperatively, regardless of EGFR mutation positivity. In addition, the molecular target therapies used in our study patients ranged from first to third generation EGFR TKIs (gefitinib, erlotinib, afatinib, and osimertinib), EGFR monoclonal antibody (cetuximab), anaplastic lymphoma kinase (ALT) inhibitors (crizotinib), mesenchymal-epithelial transition (MET) inhibitors (savolitinib, capmatinib), and PD-1 inhibitors (avelumab, nivolumab, and pembrolizumab). Therefore, it would be impossible and meaningless to stratify patients by postoperative systemic treatment, given the diversity of mechanisms and the treatment effects of these agents.

There are several limitations in the current study. First, because of its retrospective nature, selection bias regarding the inclusion and exclusion criteria cannot be ruled out. Second, there is a possibility that the differences in surgical aggressiveness between individual cases may have influenced the patients’ prognosis and survival, such as the case described in Figure 4 [21,22]. However, this possible effect of surgical strategy on patients’ outcomes was not considered in the analysis. Third, because this study included only lung adenocarcinoma patients, our results cannot be generalized to spinal metastases of various primary cancers. Finally, and most importantly, because we did not aim to develop a new prognostic model in this study and include all relevant prognostic factors in the analysis, we cannot perform any validations, including calibrations, on our results. We also cannot suggest how to incorporate biological factors into the decision-making systems as a modifier, which is well beyond the current study’s scope. Despite these limitations, the results of this study provide valuable information for state-of-the-art care for patients with spinal metastasis, and suggest future directions for the development of decision-making systems for spinal metastasis.

## 5. Conclusions

EGFR mutation positivity and the chronicity of spinal metastasis provide additional prognostic value for NSCLC patients with spinal metastasis. These results signify the importance of considering biological profiles in the decision-making process for spinal metastasis.

## Figures and Tables

**Table 1:** The New England Spinal Metastasis Score (NESMS).

Characteristics	Points Assigned
1. Modified Bauer Score	
* No visceral metastasis (1 point)*	-
* Primary tumor is not lung cancer (1 point)*	-
* Primary tumor is breast, renal, lymphoma, or myeloma (1 point)*	-
* Single skeletal metastasis (1 point)*	-
Score ≤ 2	0
Score ≥ 3	2
2. Ambulatory function	
Dependent ambulator/non-ambulator	0
Independent ambulator	1
3. Serum albumin	
<3.5 g/dL	0
≥3.5 g/dL	1

**Table 2:** Characteristics of the study cohort.

Categories	Variables	*n* (%)
Location of spinal metastasis	Cervical	16 (18.8%)
	Cervicothoracic	7 (8.2%)
	Thoracic	41 (48.2%)
	Thoracolumbar	3 (3.5%)
	Lumbar	18 (21.2%)
Chronicity of spinal metastasis	Synchronous	58 (68.2%)
	Metachronous	27 (31.8%)
EGFR mutation	Positive	38 (44.7%)
	Negative	47 (55.3%)
Ambulatory status	Independent ambulator	62 (72.9%)
	Dependent ambulator/non-ambulator	23 (27.1%)
Serum albumin	≥3.5 g/dL	67 (78.8%)
	<3.5 g/dL	18 (21.2%)
NESMS	0	8 (9.4%)
	1	25 (29.4%)
	2	52 (61.2%)

**Table 3:** Results of the multivariable Cox proportional hazards model.

Categories	Stratifications	Hazard Ratio (95% CI)	*p*-Value
NESMS	0	3.21 (1.44, 7.18)	0.0045
	1	2.57 (1.47, 4.50)	0.0010
	2	1	
Chronicity	Synchronous	1.88 (1.13, 3.12)	0.0149
	Metachronous	1	
EGFR mutation	Positive	2.10 (1.30, 3.38)	0.0024
	Negative	1	

**Table 4:** The changes in the discrimination ability (Uno’s C-index) of prognostic models by adding biological factors.

Model	Uno’s C-Index (95% CI)	*p*-Value	Adjusted *p* *
NESMS	0.59 (0.54, 0.65)		
NESMS + chronicity	0.62 (0.56, 0.69)	0.0760	0.2280
NESMS + EGFR	0.64 (0.58, 0.71)	0.0063	0.0189
NESMS + chronicity + EGFR	0.67 (0.61, 0.74)	0.0024	0.0042

* *p*-value adjusted using the Bonferroni method.

**Table 5:** The changes in the prediction ability (time-dependent area under curve (AUC)) of prognostic models by adding biological factors.

Model	Time-Dependent AUC at 6 Months (95% CI)	*p*-Value	Adjusted *p* *
NESMS	0.63 (0.53, 0.74)		
NESMS + chronicity	0.67 (0.55, 0.79)	0.1531	0.4593
NESMS + EGFR	0.69 (0.57, 0.81)	0.0320	0.0960
NESMS + chronicity + EGFR	0.73 (0.64, 0.82)	0.0073	0.0219

* *p*-value adjusted by Bonferroni method.

## Data Availability

All relevant raw data from the data presented in the manuscript or the supplementary figures and tables are available from the authors of the study upon request.
